# Percutaneous Drainage of a Post‐ERCP Liver Abscess: Successful Management of a Rare Complication—A Case Report

**DOI:** 10.1002/ccr3.71814

**Published:** 2026-01-04

**Authors:** Yavor Asenov, Georgi Jelev, Ivan Vasilev, Boris Kunev, Marin Parunev, Nikolay Penkov, Teophil Sedloev, Ivan Dimitrov

**Affiliations:** ^1^ Department of Surgery University Hospital “Tsaritsa Joanna – ISUL”, Medical University Sofia Bulgaria; ^2^ Department of Gastroenterology University Hospital “Tsaritsa Joanna – ISUL”, Medical University Sofia Bulgaria

**Keywords:** biliary stent, endoscopic retrograde cholangiopancreatography (ERCP), percutaneous drainage, post‐ERCP liver abscess, pyogenic liver abscess

## Abstract

Endoscopic retrograde cholangiopancreatography (ERCP) with sphincterotomy is an established treatment for choledocholithiasis but may rarely lead to pyogenic liver abscess (PLA). We report the case of a 72‐year‐old woman with a history of biliary stenting who presented with jaundice and cholestasis. ERCP with lithotripsy and sphincterotomy successfully cleared multiple stones, but she developed high fever and leukocytosis within 48 h. Imaging revealed a 6‐cm abscess in segment 7 of the liver. Ultrasound‐guided percutaneous catheter drainage was performed, yielding purulent material that cultured *Enterococcus faecalis* . The patient received a total of 10 days of targeted antibiotic therapy, with rapid defervescence, normalization of inflammatory markers, and ultrasound‐confirmed resolution of the abscess. She was discharged in stable condition, without recurrence. This case highlights the value of percutaneous drainage as a minimally invasive and effective management option for post‐ERCP PLA and emphasizes the importance of structured long‐term follow‐up in patients with biliary stents.

## Key Clinical Message

Liver abscess following ERCP is a rare complication that requires prompt recognition. This case demonstrates that ultrasound‐guided percutaneous drainage combined with antibiotics can lead to full recovery and avoid surgical intervention.

## 1 Introduction

Endoscopic retrograde cholangiopancreatography (ERCP) with endoscopic sphincterotomy (ES) remains the gold standard for bile duct stone management, offering effective and minimally invasive biliary decompression and stone extraction [1]. Although generally safe, ERCP with ES carries inherent risks, including rare but potentially life‐threatening complications such as pyogenic liver abscess (PLA), particularly in patients with a disrupted hepatobiliary barrier [2]. The cumulative incidence of PLA after ES has been reported as approximately 2.4% at 5 years and 3.9% at 10 years [3]. Management strategies continue to evolve, but timely diagnosis and effective source control remain essential, with minimally invasive drainage playing a central role in most patients.

Here, we report a case of a unilocular liver abscess developing after ERCP for choledocholithiasis, successfully treated with percutaneous drainage. This case highlights the interplay of pathophysiology, risk factors, and clinical decision‐making in post‐ERCP PLA and reinforces the role of percutaneous techniques as a cornerstone in the management of post‐procedural hepatobiliary infections.

## 2 Case Presentation

### 2.1 Patient History and Presenting Symptoms

We present the case of a 72‐year‐old female admitted to the Gastroenterology Clinic for evaluation of jaundice and cholestasis. Her medical history was notable for beta thalassemia, with no known family history of the disease. In 2010, she underwent laparoscopic cholecystectomy for acute calculous cholecystitis, with no evidence of choledocholithiasis at that time. In 2014, however, she developed residual choledocholithiasis complicated by cholangitis, for which she underwent lithotripsy followed by placement of self‐expanding covered biliary stents. The patient was lost to follow‐up and did not attend the scheduled examinations thereafter. According to both the patient and her relatives, she did not undergo any medical evaluations during the intervening 10‐year period, and no medical documentation was available to suggest otherwise.

On her current admission, she presented with dull, palpable pain in the epigastrium and right upper quadrant, without signs of peritoneal irritation. She was jaundiced, with a fever up to 39°C. Upon examination, she was conscious and alert, with a blood pressure of 130/70 mmHg and a heart rate of 95 beats per minute.

### 2.2 Diagnostic Workup

Laboratory tests at admission revealed leukocytosis, elevated liver enzymes, and markedly increased inflammatory markers, consistent with an active infectious process (detailed values are shown in Table 1).

**TABLE 1:** Laboratory results at admission.

Parameter	Value at admission	Normal range
Leukocytes (×10^9^/L)	15.0	4.0–10.0
ALT (U/L)	95	7–55
ALP (U/L)	136	30–120
AST (U/L)	35	10–40
GGT (U/L)	122	8–61
CRP (mg/L)	28	< 5
LDH (U/L)	403	135–225
Creatinine (μmol/L)	118	44–133
Urea (mmol/L)	11	2.5–7.8
APTT (s)	26.6	25–35
PT (%)	71	70–130
INR	1.26	0.8–1.2

Abdominal ultrasound demonstrated hepatomegaly with a hypoechoic, heterogeneous structure, irregular contours, and early caudate lobe hypertrophy. The common bile duct was dilated up to 17 mm and contained multiple stones, while the previously placed stent was not visualized. Mild intrahepatic biliary dilation was also noted.

Urgent ERCP with sphincterotomy and lithotripsy was performed, successfully extracting more than ten stones. Empirical antibiotic therapy with ciprofloxacin 400 mg IV every 12 h was initiated. Despite this, the patient remained febrile, with temperatures up to 40°C and worsening leukocytosis (29 × 10^9^/L).

**FIGURE 1:**
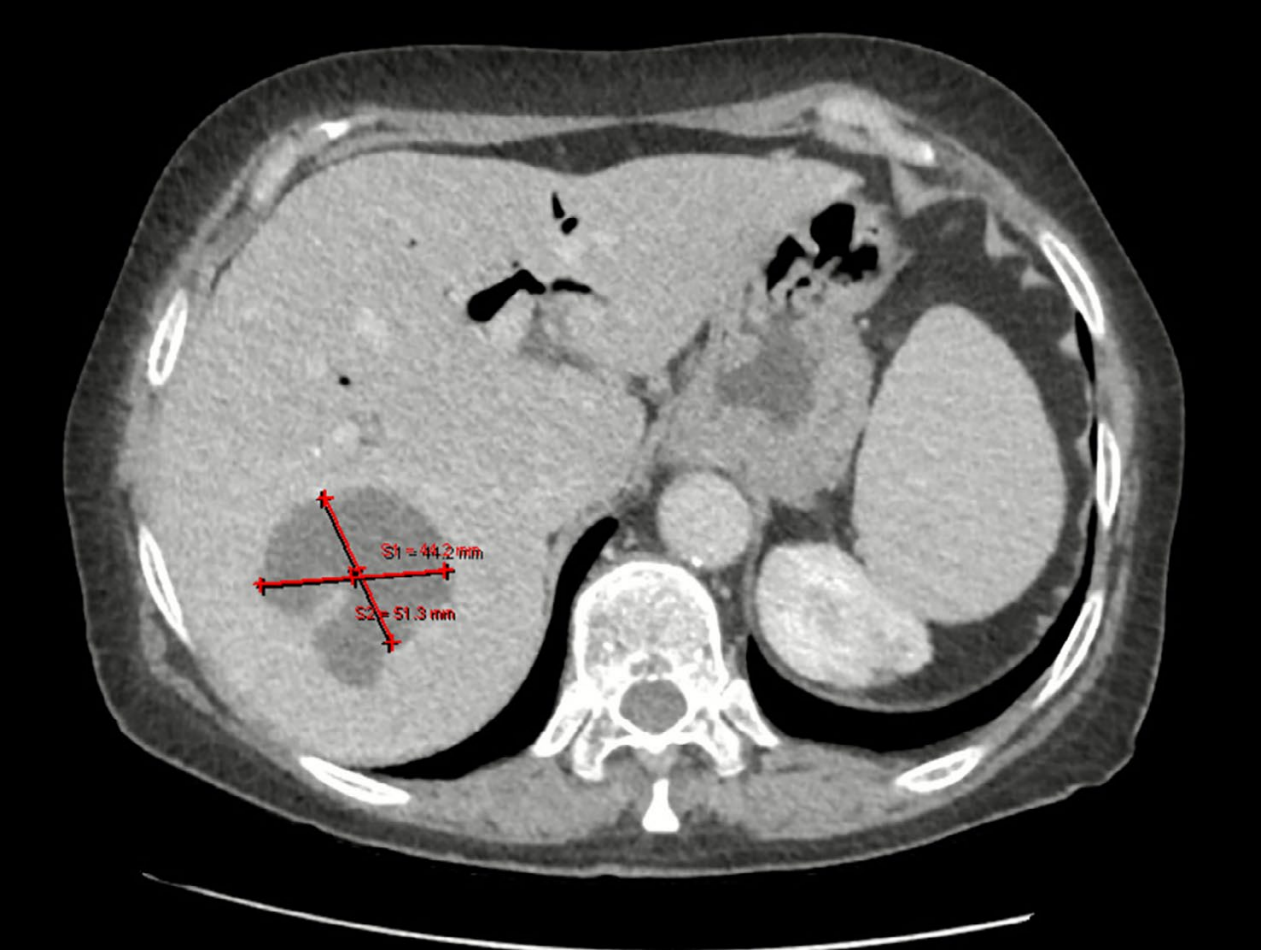
Contrast‐enhanced CT scan of the abdomen showing a pyogenic liver abscess in the right lobe.

On post‐procedural day 2, repeat ultrasound revealed moderate pneumobilia and a multiloculated, heterogeneous, predominantly anechoic lesion in the right liver lobe measuring approximately 6 cm, raising suspicion of a liver abscess. Contrast‐enhanced CT confirmed a hypodense lesion measuring 51 mm in segments 6 and 7, with gas inclusions and peripheral enhancement, consistent with a pyogenic abscess (Figure 1).

### 2.3 Therapeutic Intervention

The patient was transferred to the Surgery Clinic, where percutaneous drainage under ultrasound guidance was performed on post‐ERCP day 3. Premedication consisted of 1000 mg metamizole (Analgin) and 100 mg tramadol (Lydol) intramuscularly. Local anesthesia was achieved with 30 mL of 1% lidocaine.

**FIGURE 2:**
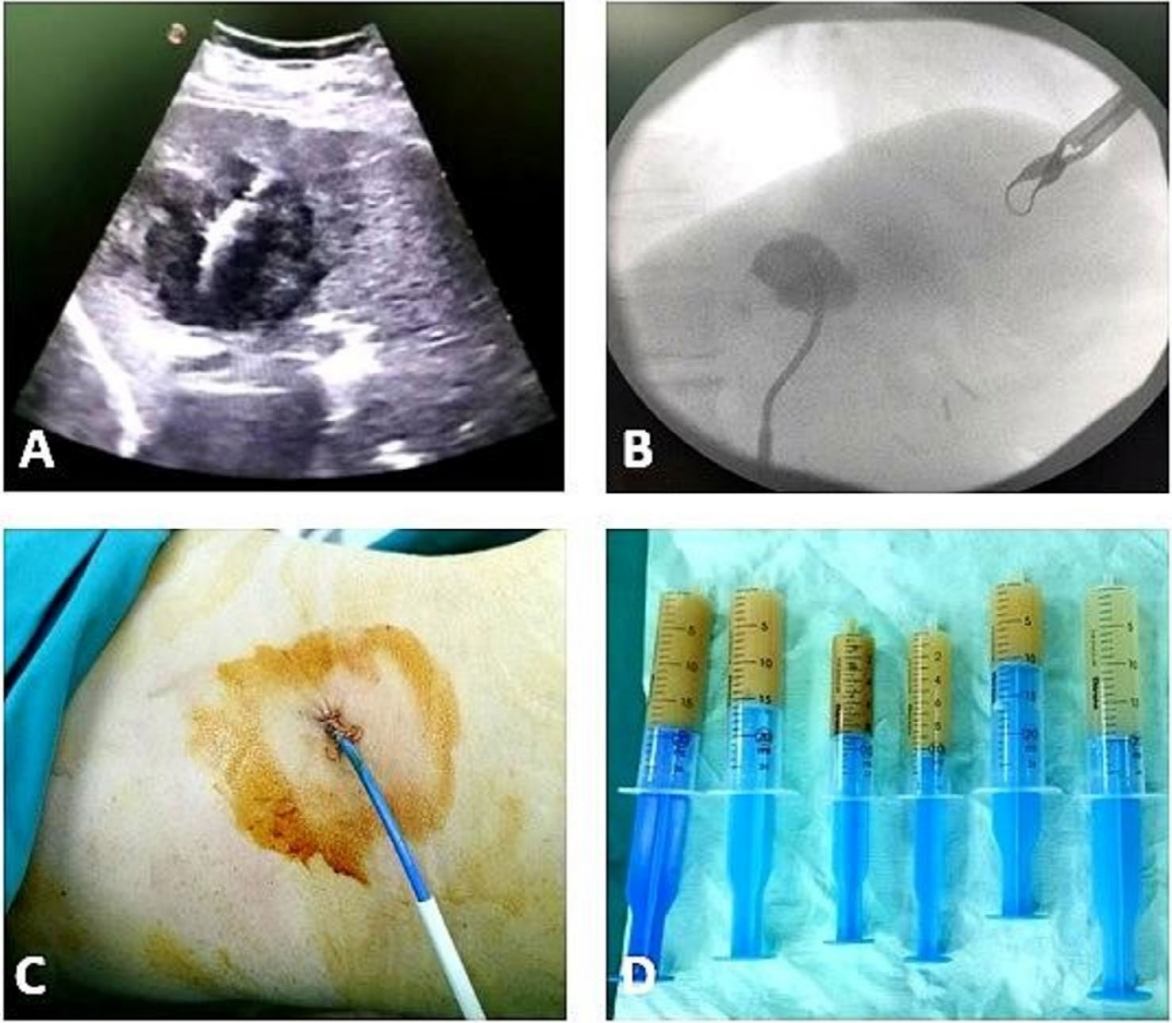
Percutaneous drainage procedure for liver abscess. (A) Ultrasound‐guided positioning of the needle within the abscess cavity. (B) Fluoroscopic imaging confirming the correct placement of the drainage catheter. (C) External appearance of the drainage catheter secured in place. (D) Aspirated purulent material collected during the procedure.

Under real‐time ultrasound guidance, an 18G needle was introduced into the abscess cavity, followed by placement of a 0.035‐in. guidewire. Stepwise dilation was carried out, and a 10 Fr pigtail catheter was inserted and secured, allowing continuous drainage (Figure 2).

### 2.4 Outcome and Follow‐Up

Clinical improvement was rapid, with fever resolution within 24 h and gradual normalization of inflammatory markers.

Microbiological culture of the drained pus yielded *Enterococcus faecalis* , sensitive to fluoroquinolones, ampicillin, and vancomycin. Given susceptibility to ciprofloxacin, the empirical regimen was continued without modification. Antibiotics were administered for a total of 10 days (2 days pre‐drainage, the day of the drainage procedure, and 7 days post‐drainage).

**FIGURE 3:**
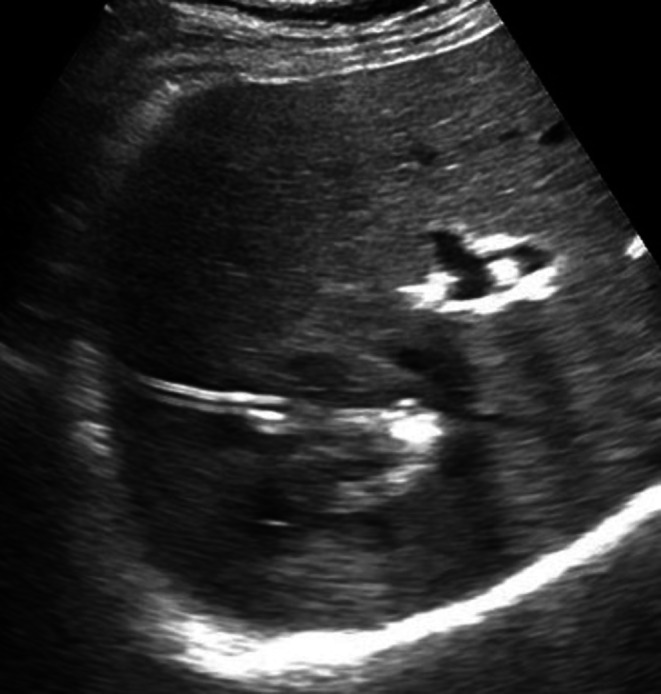
Post‐procedural ultrasound on day 3 confirming the correct positioning of the drainage catheter and resolution of the abscess cavity.

The catheter was flushed daily with saline to maintain patency. Drain output decreased from purulent fluid on day 1 to serous by day 3, with daily volume falling below 20 mL by day 3 and remaining < 10 mL/24 h for the next days. Daily ultrasound monitoring confirmed progressive cavity collapse and correct catheter position. (Figure 3).

On postoperative day 7, meeting criteria of output < 10 mL/24 h, clinical resolution, normalization of inflammatory markers, and imaging evidence of cavity reduction, the drain was removed. The patient was discharged in stable condition with complete recovery and no recurrence of fever or infection.

## 3 Discussion

Disruption of the hepatobiliary barrier after sphincterotomy facilitates duodenal–biliary reflux, bacterial colonization, and recurrent cholangitis, while additional risk factors such as diabetes, biliary obstruction, and malignancy further increase susceptibility [3, 4]. Large population studies confirm the higher long‐term risk of PLA after ES and also suggest an association with underlying gastrointestinal malignancy, particularly in the presence of choledocholithiasis [3, 4]. Biliary stent placement, though widely used, carries its own risks: incidence rates of PLA range from 4.3% to 13.5%, with mortality reported up to 30% [5]. Contributing mechanisms include bile stasis, retrograde infection, stent‐related trauma, and biofilm formation [6, 7].

In our patient, several of these risk factors converged. Prior sphincterotomy had permanently weakened the hepatobiliary barrier, allowing duodenal–biliary reflux and colonization. The long‐standing, unmonitored stent served as a nidus for biofilm formation and chronic microbial persistence, while choledocholithiasis and bile duct dilatation promoted stasis. Recent metagenomic studies confirm that biofilms on biliary stents are enriched with virulence and resistance determinants, and prolonged or “forgotten” stents are strongly associated with recurrent cholangitis and septic complications [6, 7, 8]. The absence of an abscess on pre‐procedural ultrasound supports a periprocedural onset, precipitated by repeat ERCP in an already compromised biliary system.

While Gram‐negative bacilli such as *Escherichia coli* and *Klebsiella pneumoniae* remain the predominant pathogens in PLA, Gram‐positive cocci—particularly *Enterococcus* spp.—are increasingly reported, especially in post‐ERCP cases [9, 10]. These organisms exhibit intrinsic resistance to cephalosporins, which are still commonly used for prophylaxis and empiric therapy in biliary infections; prior cephalosporin exposure further increases this risk [9]. For *E. faecalis* , ampicillin or ampicillin/sulbactam is preferred when susceptible, piperacillin–tazobactam provides reliable empiric coverage, and glycopeptides are reserved for resistance or allergy [11, 12]. In our case, the isolate was *E. faecalis* , resistant to ceftriaxone but susceptible to fluoroquinolones. Accordingly, culture‐directed ciprofloxacin was continued, consistent with guideline recommendations that optimal management integrates targeted antimicrobial therapy with timely abscess drainage [12, 13].

Historically, antibiotic courses for PLA extended for several weeks, with a pooled mean of ~33 days across heterogeneous series [14]. More recent evidence and international guidelines support shorter regimens—approximately 4–7 days after adequate drainage and clinical improvement—with therapy de‐escalated once culture results are available [12, 13]. Studies in cholangitis further confirm that abbreviated therapy is non‐inferior when source control is achieved [15, 16]. Our patient improved promptly after drainage, with resolution of fever, normalization of inflammatory markers, and collapse of the abscess cavity. Ciprofloxacin was continued for a total of 10 days, a reasonable choice given the susceptibility profile, rapid recovery, and alignment with contemporary short‐course recommendations [12, 13, 14].

Early recognition and prompt management of PLA are critical, as delayed diagnosis can lead to septic shock, multiorgan failure, and death [10]. Advances in imaging and interventional radiology have made percutaneous drainage the first‐line therapy, typically indicated for abscesses > 5 cm, persistent fever despite 48–72 h of antibiotics, or imaging features suggesting impending perforation [10]. Reported success rates exceed 80%–90%, although 8%–36% of patients may still require surgical intervention, which today is largely reserved for multiloculated, ruptured, or refractory abscesses [10, 17].

Percutaneous catheter drainage (PCD) consistently outperforms needle aspiration (PNA). Success rates approach 90% compared with 70%–80% for PNA, with faster clinical improvement (≈4 vs. 7 days), earlier 50% cavity shrinkage (≈4 vs. 7 days), and slightly shorter hospital stays (≈11 vs. 13 days). Continuous drainage therefore ensures more reliable resolution, particularly in larger or partially liquefied abscesses [18, 19].

EUS‐guided drainage is an emerging option, particularly for abscesses in the left lobe or caudate adherent to the gastric or duodenal wall. Technical success rates of 97%–100% with low complication rates have been reported, and comparative studies suggest faster resolution and shorter hospital stay than PCD in selected cases [20, 21]. For right‐lobe disease, however, feasibility remains limited: one recent series achieved ~79% technical and 93% clinical success, underscoring both the potential and the expertise‐dependent nature of this approach [22].

Percutaneous drainage, by contrast, benefits from decades of accumulated experience and remains the most established and versatile option, with proven efficacy across all lobes and in complex or multiloculated abscesses where multiple catheters or lavage may be required. EUS‐guided drainage is best regarded as complementary, with the choice between approaches determined by abscess location, complexity, expertise, and institutional resources. In our patient, the subdiaphragmatic segment 7 abscess was anatomically remote from the stomach and duodenum, making EUS less feasible. PCD provided safe access, continuous drainage, and daily monitoring, leading to both clinical and radiological resolution.

## 4 Conclusion

This case illustrates how multiple risk factors—prior sphincterotomy, long‐standing biliary stenting, and choledocholithiasis—can converge to precipitate post‐ERCP pyogenic liver abscess. The successful management reinforces evidence from the literature supporting percutaneous drainage as the cornerstone of treatment, with EUS‐guided drainage emerging as a valuable complementary option in selected patients. This reflects the broader shift in liver abscess management, where minimally invasive strategies are replacing surgery as the first‐line approach, with operative intervention now reserved for refractory or complex cases.

## Author Contributions

**Yavor Asenov:** methodology, writing – original draft. **Georgi Jelev:** conceptualization. **Ivan Vasilev:** validation, visualization. **Boris Kunev:** formal analysis, project administration. **Marin Parunev:** investigation. **Nikolay Penkov:** data curation. **Teophil Sedloev:** resources. **Ivan Dimitrov:** supervision, writing – review and editing.

## Funding

The authors have nothing to report.

## Disclosure

The authors have nothing to report.

## Ethics Statement

This case report was conducted in accordance with institutional standards for clinical case reporting. Ethics committee approval was not required for single‐patient case reports at our institution.

## Consent

Written informed consent was obtained from the patient to publish this case report in accordance with the journal's patient consent policy.

## Conflicts of Interest

The authors declare no conflicts of interest.

## Data Availability

The data that support the findings of this study are available upon request from the corresponding author.
